# Managing Mesenteric-Side Small Bowel Perforation in the Setting of Non-ST Elevation Myocardial Infarction: A Dual Challenge

**DOI:** 10.7759/cureus.49226

**Published:** 2023-11-22

**Authors:** Mena Louis, Matthew Vassy, Claudia Gherasim, Hardeep Singh

**Affiliations:** 1 General Surgery, Northeast Georgia Medical Center Gainsville, Gainesville, USA; 2 Trauma and Acute Care Surgery, Northeast Georgia Medical Center Gainsville, Gainesville, USA; 3 Pathology, Northeast Georgia Medical Center Gainsville, Gainsville, USA; 4 Research, Northeast Georgia Medical Center Gainsville, Gainsville, USA

**Keywords:** small bowel resection, laparotomy, mesentery, nstemi, small bowel perforation

## Abstract

Small bowel perforations are critical surgical emergencies, and those occurring on the mesenteric side are particularly uncommon. These perforations can lead to significant morbidity due to potential vascular compromise and the rapid spread of intraluminal contents. When a patient concurrently presents with a non-ST elevation myocardial infarction (NSTEMI), the clinical management becomes even more intricate. Balancing the urgency of surgical intervention for bowel perforation with the potential cardiac risks associated with surgery, especially in the context of a concurrent NSTEMI, poses a significant clinical challenge. An 86-year-old male with an extensive cardiac history presented with a complaint of abdominal pain, primarily localized to the left lower quadrant. Diagnostic investigations, including a contrast-enhanced computerized tomography (CT) scan, identified extraluminal air and pronounced inflammation adjacent to a loop of small bowel, consistent with perforation. Simultaneously, elevated troponin levels and specific electrocardiogram (ECG) changes confirmed an NSTEMI diagnosis. Following a multidisciplinary discussion, the patient underwent exploratory laparotomy, resulting in small bowel resection. Postoperative cardiac monitoring managed a brief episode of supraventricular tachycardia effectively. This case highlights the intricacies involved in managing a patient with a rare mesenteric-side small bowel perforation while also dealing with an NSTEMI. While the causes of spontaneous small bowel perforations can vary, this case presented an added layer of complexity without a clear predisposing factor. The presence of NSTEMI introduced challenges in determining the timing and approach to surgical intervention. The necessity for collaboration between surgical and cardiology teams was evident, ensuring a comprehensive assessment of the patient's cardiac risk and optimizing cardiac medications. Managing a patient with concurrent small bowel perforation and NSTEMI demands meticulous clinical judgment and inter-specialty collaboration. This case offers valuable insights into the considerations and challenges faced in such unique clinical scenarios, emphasizing the importance of individualized patient care.

## Introduction

Small bowel perforations, particularly on the mesenteric side, are a rare but serious surgical emergency, often presenting with non-specific clinical symptoms [1,2]. The diagnosis and management of such cases require a high index of clinical suspicion and timely intervention to prevent associated morbidity and mortality [3]. Concurrent presentation with non-ST elevation myocardial infarction (NSTEMI) further complicates the clinical scenario. NSTEMI, characterized by myocardial ischemia without ST-segment elevation on the electrocardiogram (ECG), necessitates its own set of diagnostic and management strategies, which may conflict with the urgent needs of managing bowel perforation [3]. The intersection of these two conditions creates a significant medical challenge. On the one hand, small bowel perforation requires immediate surgical intervention to mitigate the risk of sepsis and potential mortality [3,4]. On the other hand, NSTEMI necessitates cardiac stabilization and may delay surgical intervention due to the elevated risk of myocardial injury during surgery [5]. Therefore, each condition presents contrasting imperatives that make simultaneous management both urgent and precarious.

This case report presents an 86-year-old male patient who arrived with a rare spontaneous mesenteric-side small bowel perforation alongside signs of an NSTEMI, underscoring the diagnostic challenges and management intricacies involved in such complex clinical presentations.

## Case presentation

An 86-year-old male with a notable cardiac history, including coronary artery disease (CAD) status post-coronary artery bypass grafting (CABG) 13 years prior, recent successful intravascular ultrasound (IVUS) guided percutaneous coronary intervention/drug-eluting stent (PCI/DES) of a severe calcific ostial right coronary artery (RCA), hypertension, aortic stenosis (AS), hyperlipidemia, and type 2 diabetes, presented to the Emergency Department (ED) with abdominal pain persisting for several days. The pain was predominantly localized to the left lower quadrant region and radiating throughout the abdomen. The patient denied any gastrointestinal symptoms such as nausea, vomiting, or diarrhea, as well as any urinary complaints. Additionally, there were no reports of chest pain, shortness of breath, or exertional dyspnea. Initial labs in the ED were significant for hypokalemia, leukocytosis, and elevated high-sensitivity troponin (Table 1).

**Table 1:** Initial labs on presentation

Lab Test	Patient's Value	Normal Range
Sodium (Na)	140 mmol/L	135-148 mmol/L
Potassium (K)	3.3 mmol/L	3.5-5.2 mmol/L
Creatinine	1.3 mg/dL	0.80-1.30 mg/dL
Glucose	158 mg/dL	65-99 mg/dL
Total bilirubin	1.2 mg/dL	0.00-1.00 mg/dL
High-sensitivity troponin I	812 pg/mL	<14 pg/mL
White blood cell (WBC) count	13.8 K/µL	4.8-10.8 K/µL

Diagnostic workup

**Figure 1:**
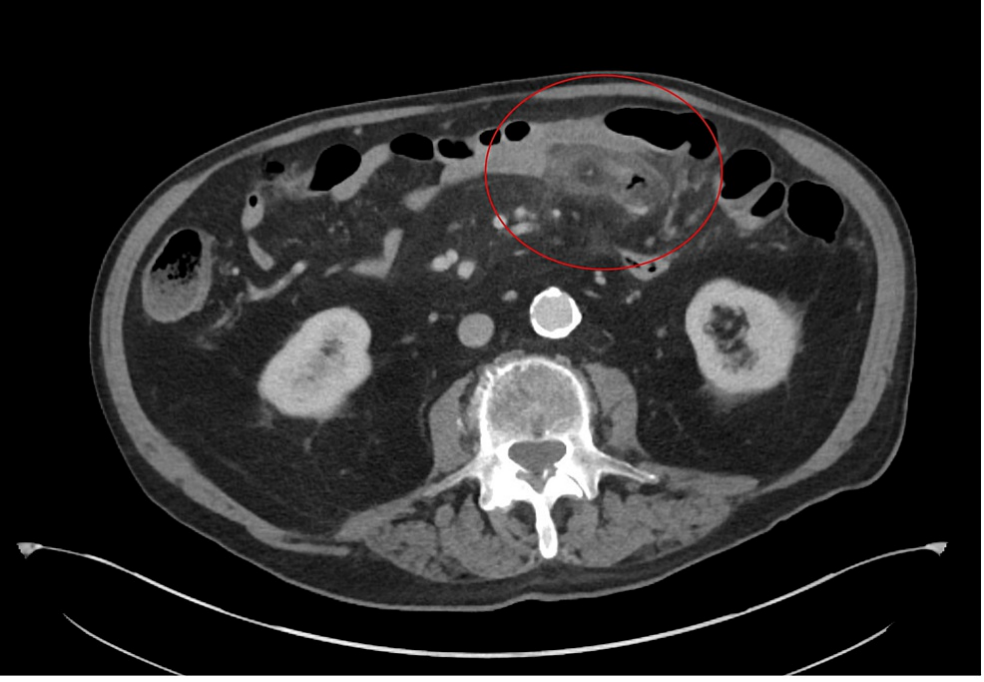
Axial CT image. In the left side of the abdomen, there is a perforation (red circle), which appears to involve a small bowel with extraluminal air and an intense inflammatory reaction.

**Figure 2:**
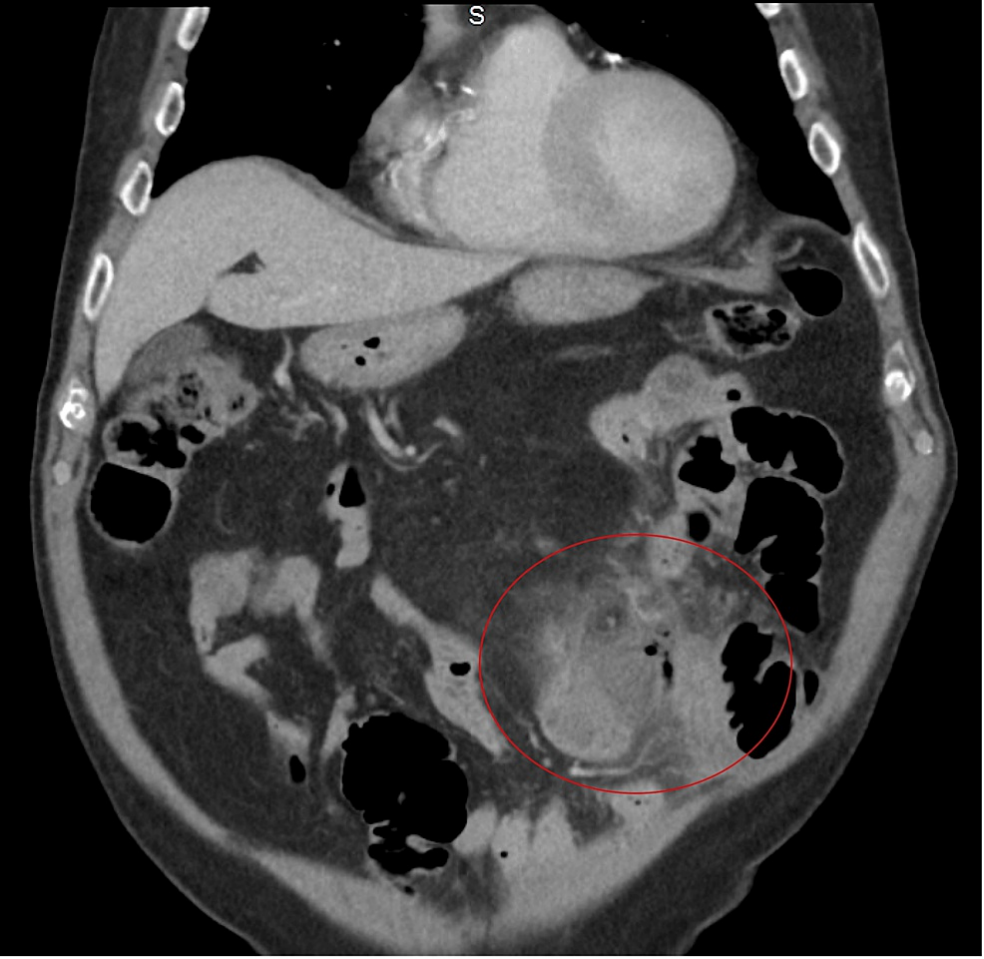
A coronal CT image showing bowel perforation on the left side of the abdomen (red circle). This appears to involve the small bowel, with surrounding inflammatory reaction and extraluminal air, but there is no evidence of a definable abscess, and no distant free intraperitoneal air is visible.

In the ED, a contrast-enhanced computed tomography (CT) scan of the abdomen and pelvis was performed, revealing findings indicative of bowel perforation. Notably, the CT scan demonstrated a perforation on the left side of the abdomen, suspected to be of the small bowel, indicated by extraluminal air and a pronounced inflammatory response. This perforation did not appear to involve the colon, and there was no evidence of a distinct abscess. Adjacent to the perforation site, mild dilatation of the small bowel was observed, while the more distal small bowel segments appeared non-dilated (Figures 1, 2).

**Figure 3:**
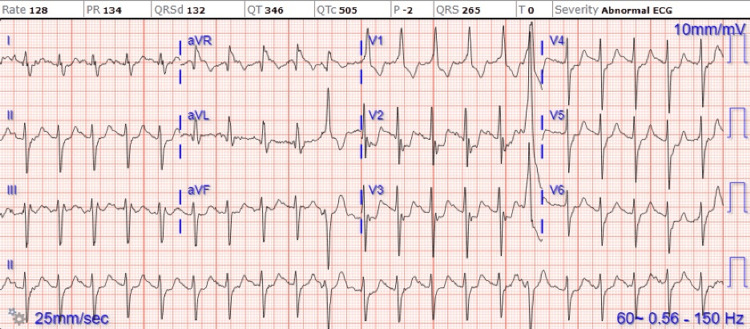
Initial ECG on admission showing sinus tachycardia, premature atrial complexes with aberrant conduction, right bundle branch block, atrial premature complexes, and aberrant conduction of supraventricular beats.

Laboratory investigations revealed a high-sensitivity troponin I level of 812, and an electrocardiogram (ECG) displayed sinus tachycardia with frequent premature atrial contractions (PACs) and non-significant ST-T wave changes (Figure 3). Given the elevated troponin levels, the patient was initiated on intravenous heparin in the ED, and cardiology was promptly consulted.

A transthoracic echocardiogram (TTE) was performed, revealing a normal-sized left ventricle with normal wall thickness. The septal motion was consistent with a bundle branch block, and the left ventricular systolic function was normal with an estimated ejection fraction (EF) of 50-55%. The aortic valve (AV) showed moderately calcified cusps and mild to moderate stenosis. The aortic valve mean gradient is 16 mmHg. AV peak gradient is 27 mmHg. AV peak velocity is 2.6 m/s.

Clinical management

Upon further evaluation in the Emergency Department, the patient appeared tachycardic and ill, with diffuse abdominal tenderness suggestive of peritonitis. The etiology of the perforation remained uncertain at this juncture. After discussing the potential risks, benefits, and outcomes of surgical intervention, the patient was informed of the suspected small bowel perforation and the associated risks of sepsis and potential mortality. The patient comprehended the situation and consented to proceed with the recommended surgical intervention.

The patient's cardiac history, particularly the concurrent NSTEMI diagnosis, posed additional challenges. The patient had a history of multiple CABG and PCI procedures and was on dual antiplatelet therapy (DAPT). Given the surgical requirements, oral antiplatelets were temporarily withheld.

Surgical intervention

**Figure 4:**
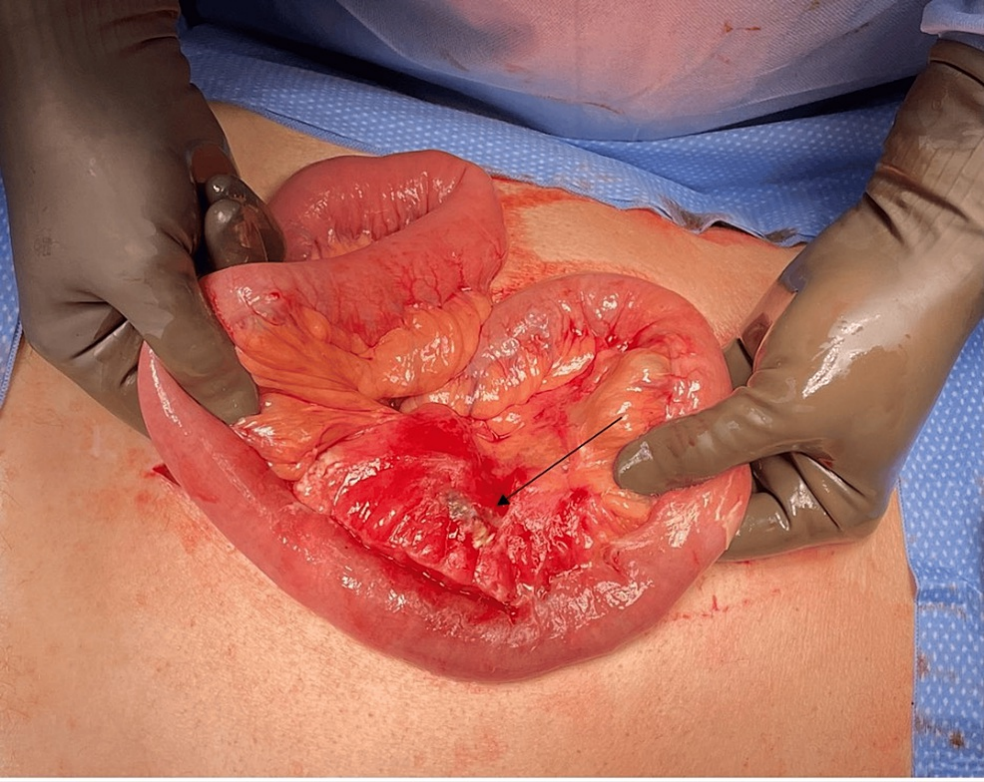
Intraoperative gross picture showing an area of inflammatory changes involving primarily the mesentery, which represented a small bowel perforation into the mesentery (black arrow).

The patient underwent an exploratory laparotomy, during which a segment of the small bowel, approximately 60 cm from the ligament of Treitz, displayed significant inflammatory changes, primarily involving the mesentery (Figure 4). This segment, measuring about 20 cm in length, appeared inflamed and edematous, with necrotic mesentery. The affected segment was resected, and an anastomosis was performed. Intraoperative SPY fluorescence imaging with indocyanine green (ICG) was utilized to ensure adequate perfusion of the resected margins.

Postoperative course

Following surgery, cardiology continued to monitor the patient for the NSTEMI. The heparin drip was resumed postoperatively, and clopidogrel (Plavix) was temporarily withheld. The patient remained nothing per mouth (NPO) immediately after surgery but was advanced to a cardiac low-fat diet on postoperative day 1. On the second postoperative day, the patient experienced chest pain and supraventricular tachycardia (SVT) overnight, necessitating the initiation of an Amiodarone infusion. By the third postoperative day, the Amiodarone infusion was discontinued, and the patient's diet was advanced to regular consistency. The patient reported multiple bowel movements by the fourth postoperative day. On the fifth postoperative day, clopidogrel was resumed. The patient was subsequently discharged with instructions to follow-up with the surgeon, primary care physician, and cardiologist.

Pathology

**Figure 5:**
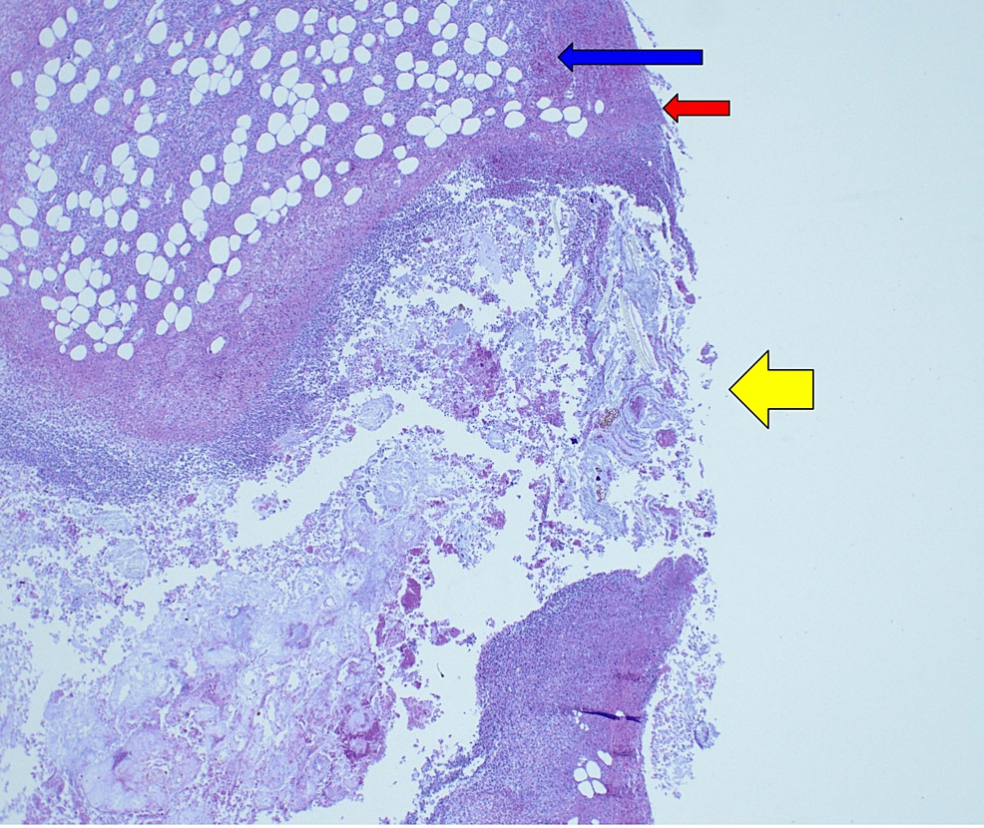
4× magnification view showing small bowel mesentery (blue arrow) with mesothelial lining of peritoneal surface (red arrow). The yellow arrow points to a perforation tract on the small bowel mesentery side.

The pathology of the resected small bowel segment revealed acute serositis, indicative of inflammation of the serosal surface. Notably, the primary segment of the specimen displayed pronounced transmural inflammation, characterized by both acute and chronic inflammatory changes (Figure 5). This extensive inflammation had led to the formation of an abscess, which subsequently resulted in a perforation. Importantly, the resection margins were found to be viable, suggesting healthy tissue boundaries. Furthermore, the entire examined specimen showed no evidence of tumor presence.

## Discussion

Small bowel perforations, while rare, present significant clinical challenges due to their potential for rapid progression and associated morbidity [6]. The small bowel, given its length and mobility, can perforate at various sites. However, perforations on the mesenteric side are particularly uncommon. The mesentery, responsible for the vascular supply and lymphatic drainage of the intestine, is crucial for bowel viability. A perforation at this site can lead to vascular compromise, rapid spread of intraluminal contents, and severe localized inflammation, resulting in peritonitis [7].

The etiology of spontaneous small bowel perforations is diverse. Common causes include nonsteroidal anti-inflammatory drug (NSAID) use, infections, malignancies, and inflammatory conditions such as Crohn's disease [2,6]. However, a spontaneous perforation, especially at the mesenteric side without a clear predisposing factor, remains a diagnostic challenge [1,2]. In this case, the exact cause of the perforation remains undetermined, highlighting the importance of a thorough diagnostic workup and the potential for unexpected findings in clinical practice.

Concurrent with the bowel perforation, the patient presented with an NSTEMI [8]. NSTEMIs are part of the acute coronary syndrome spectrum and are characterized by myocardial ischemia without ST-segment elevation on the ECG [4]. The primary management goals for NSTEMI include stabilizing the cardiac condition, risk stratification, and timely revascularization [5,9]. The presence of elevated troponin levels, as seen in this patient, is a key diagnostic marker for myocardial injury and necessitates prompt cardiology consultation and intervention [9].

The coexistence of small bowel perforation and NSTEMI in a patient introduces a myriad of management complexities [6,10]. Surgical intervention for bowel perforation is urgent, given the risk of sepsis and potential mortality [7,11]. However, the concurrent NSTEMI complicates the surgical decision-making process [1,2]. Surgery, especially major procedures like laparotomy, poses a risk of cardiac complications, including myocardial infarction, arrhythmias, and even cardiac arrest. Balancing the urgency of surgical intervention for bowel perforation with the potential cardiac risks associated with surgery becomes paramount [12,13].

In the present case, the management of NSTEMI pre-operatively involved a balancing act. Immediate cardiology consultation was sought, and the patient was initiated on intravenous heparin to stabilize the cardiac condition. However, the necessity for surgery presented a dilemma, particularly regarding the management of the patient's DAPT. The decision to temporarily withhold oral antiplatelets was taken to minimize surgical bleeding risks, but this also posed a potential risk for stent thrombosis or recurrent cardiac events.

The patient also had aortic stenosis with calcified cusps and an AV mean gradient of 16 mmHg. Aortic stenosis poses additional challenges during the induction of general anesthesia, as it can lead to abrupt changes in cardiac loading conditions. Anesthetic agents that cause vasodilation or a decrease in myocardial contractility were avoided as they can exacerbate the hemodynamic instability in patients with aortic stenosis, making anesthetic management particularly intricate in this case.

Postoperatively, cardiac monitoring was heightened. The patient experienced an episode of SVT, necessitating the initiation of an amiodarone infusion, which was later successfully discontinued. Clopidogrel was resumed on the fifth postoperative day after assessing the patient's hemostatic status and perceived cardiac risk. This highlights the need for vigilant cardiac monitoring and medication management in the perioperative period, especially in the presence of a concurrent NSTEMI.

In this case, a multidisciplinary approach was essential. Collaboration between the surgical, cardiology, and anesthesiology teams allowed for a comprehensive assessment of the patient's cardiac risk, optimization of cardiac medications, and careful timing of surgical intervention. The decision to temporarily withhold antiplatelet therapy in the perioperative period, given the patient's recent NSTEMI and history of coronary interventions, was crucial to minimize bleeding risks during surgery [12,13]. However, this also posed a potential risk of stent thrombosis or recurrent cardiac events. The decision to resume antiplatelet therapy postoperatively was based on a careful assessment of the patient's hemostatic status and the perceived cardiac risk.

## Conclusions

This case report discusses a complex clinical scenario involving an 86-year-old male patient with a rare mesenteric-side small bowel perforation, occurring concurrently with an NSTEMI. One of the critical lessons learned from this case is the need for a high index of clinical suspicion, especially when patients present with non-specific symptoms such as generalized abdominal pain. Imaging, particularly contrast-enhanced CT scans, plays a pivotal role in arriving at an accurate diagnosis and should be utilized promptly.

Another key takeaway is the importance of immediate consultation with cardiology specialists when faced with indicators of myocardial injury, such as elevated troponin levels and specific ECG changes. This is crucial even when classic cardiac symptoms are absent, as was the case here. Managing such complex cases necessitates a multidisciplinary approach. Coordination between surgical, anesthesia, and cardiology teams is essential to balance the urgency of surgical intervention against potential cardiac risks. This was particularly evident in the perioperative period, where medication management and cardiac monitoring required a tailored approach based on the patient's individual risk profile.
